# Consequences of inequity in the neurosurgical workforce: Lessons from traumatic brain injury

**DOI:** 10.3389/fsurg.2022.962867

**Published:** 2022-09-01

**Authors:** Shivani Venkatesh, Marcela Bravo, Tory Schaaf, Michael Koller, Kiera Sundeen, Uzma Samadani

**Affiliations:** ^1^ Department of Bioinformatics and Computational Biology, University of Minnesota, Minneapolis, MN United States; ^2^ Surgical Services, Minneapolis VA Medical Center, Minneapolis, MN United States

**Keywords:** sex, race, mortality, concussion, clinical research, machine learning traumatic brain injury, inequity

## Abstract

Women and minorities leave or fail to advance in the neurosurgical workforce more frequently than white men at all levels from residency to academia. The consequences of this inequity are most profound in fields such as traumatic brain injury (TBI), which lacks objective measures. We evaluated published articles on TBI clinical research and found that TBI primary investigators or corresponding authors were 86·5% White and 59·5% male. First authors from the resulting publications were 92.6% white. Most study participants were male (68%). 64·4% of NIH-funded TBI clinical trials did not report or recruit any black subjects and this number was even higher for other races and the Hispanic ethnicity. We propose several measures for mitigation of the consequences of the inequitable workforce in traumatic brain injury that could potentially contribute to more equitable outcomes. The most immediately feasible of these is validation and establishment of objective measures for triage and prognostication that are less susceptible to bias than current protocols. We call for incorporation of gender and race neutral metrics for TBI evaluation to standardize classification of injury. We offer insights into how socioeconomic factors contribute to increased death rates from women and minority groups. We propose the need to study how these disparities are caused by unfair health insurance reimbursement practices. Surgical and clinical research inequities have dire consequences, and until those inequities can be corrected, mitigation of those consequences requires system wide change.

## Introduction

Neurosurgery is the discipline of medicine that is most acutely involved in the care of brain injured patients, and it is among the least diverse of all medical specialties, rivaled most closely by orthopedics and cardiac surgery. Women represent approximately 6% (*n* = 259/4,178) (1) and black neurosurgeons represent approximately 4% (*n* = 183/4,178) (2) of all board-certified neurosurgeons in the United States (3). Lack of mentorship for junior female and minority surgeons remains an issue as there are only 33 female full professors of neurosurgery in the United States (1) (4% of the field), and an unknown number of black full professors of neurosurgery. Retention of both female and minority talent in neurosurgery remains a significant problem. Women achieve board certification at a rate between 63% and 70% (4, 5) while men are certified at a rate of 81% (4) which effectively prevents the number of female mentors from increasing commensurately with the number of female residents in training. Data on minority attrition in neurosurgery is not currently available. These workforce inequities have grave consequences for patients.

This paper examines gender and racial disparities in the field of traumatic brain injury (TBI) as a model for understanding the consequences of an inequitable workforce. Women and people of color are more likely to sustain a violent TBI (6, 7) but less likely to seek care (8). They are more likely to receive less aggressive care than others (9) and be assessed by a trainee rather than a credentialed physician (10). They are less likely to participate in post-injury rehabilitation (11) or enter a clinical trial (7, 12–14), perhaps due to reasons ranging from historical abuse, socioeconomic and education status, along with reluctance to trust a medical research system that does not treat even its own minority members equitably (15–19). People of color are up to twice as unlikely to survive their brain injury than people who are white (10, 20–23). Those who do survive an initial brain injury are more likely to commit suicide after “recovery” (21, 24) than their white counterparts.

How studies are constructed and whether they specifically analyze factors such as sex or race in outcomes is known to significantly impact the validity and applicability of the data. When studies fail at this, there is less chance for systemic improvement and improved patient outcomes. We present an analysis of TBI studies to identify opportunities for equitable improvements in the care of brain injured people. This approach was chosen to identify problems that collectively reflect deficits present in large systems rather than at a single institution or hospital center.

## Methods

### Identifying NIH funded TBI clinical trials

While ClinicalTrials.gov included NIH funding as a data point, NIH funding was also cross checked using the NIH RePORTER database. Within this module, a query was executed for TBI clinical studies using the Text Search filter and Fiscal Year filter. The following logic statement was used for the text search: “TBI” OR “traumatic brain injury” OR “head injury” OR “concussion” OR “brain injury”. All fiscal years, from Active Projects through 1985, were selected under the fiscal year project. The NIH RePORTER query resulted in 24,580 projects and 659 clinical studies. After manual review of all the clinical studies, only 69 of the trials were determined to be true TBI clinical trials. Gender and race was assigned to TBI primary investigators and authors using publicly available information provided by the author and/or investigator based on their employers websites and relevant public databases.

### Determining publication status

Publication status and publication date was determined by manually searching ClinicalTrials.Gov and PubMed. Each trial was reviewed on ClinicalTrials.gov by querying ClinicalTrials.gov with the NCT number of the trial. If there was a publication of results listed for the trial, the study was considered to be positive for having a publication. If a publication of results was not listed in the trial, the NCT number was then queried on PubMed. If the query resulted in publications, the publications listed were then reviewed to ensure they were publishing results of the clinical trial, rather than just referencing it. For example, a published paper may reference an ongoing study whose results may be interesting to the authors of the paper. In this case, the publication would not be considered as a positive publication of that study. If no publications resulted from the NCT trial number query, PubMed was queried with each investigator name listed on ClinicalTrials.gov. The following query was used on PubMed: “investigator first name and last name [au]”. All papers of the investigator from the most recent back to the clinical trial start date being searched was reviewed to determine if there was a publication of the trial results.

### Analysis

The following variables listed in ClinicalTrials.gov were analyzed: trial status, if study results were posted, intervention category, trial phase, NIH funding, and age of trial participants. The following variables extracted from ACCT database were also used in the analysis: months from primary completion date to results first posted on ClinicalTrials.gov and result reference posting. Continuous results were reported as mean ± standard deviation. Discrete results were reported as numbers and % of total. Means from 2 samples were compared by *t*-test. Proportions for discrete variables were analyzed by *χ*^2^ tests.

Probability of publication was modeled with a logistic regression using variables that were found to be significant per the *t*-test or the chi-squared test. A second logistic regression with less explanatory variables was subsequently used. In the second regression, only the enrollment variable and the variables noted as being significant in the first regression model were used. For all analyses, missing values were dropped. Twelve observations had extreme outliers, values greater than the 99th percentile, in the enrollment variable. In cases where the enrollment variable was analyzed, those seven observations were dropped.

## Results

### Identified racial and gender disparities in studies

The National Institute of Health (NIH)-funded TBI clinical trials are predominantly conducted by white men and disproportionately enroll white and male populations (Table 1) (14). TBI primary investigators or corresponding authors were 86·5% White and 59·5% male. Based on associations and available data, we estimate that the first authors from the resulting publications were 92.6% White, with gender undetermined due to lack of publicly available information.

**Table 1:** Demographic reporting from NIH funded TBI clinical trials.

Race/Ethnicity/Gender	Black	American Indian or Alaska Native	Asian	Native Hawaiian	Two or more races	His panic	White	Other or Minority group	Male	Female
Count (*n*) from NIH-funded TBI trials	638	24	51	22	44	688	4190	1452	7913	3731
% Race/Ethnicity/Gender	9·9	0·4	0·8	0·3	0·7	10·7	65·3	22·6	68·0	32·0
% Demographics of TBI patients seen in trauma center	12·3	0·0	0·0	0·0	0·0	11·3	63·1	8·0	63·9	36·1
% Demographics of TBI mortality	19·0	0·0	0·0	0·0	0·0	13·0	52·0	5·0	73·0	27·0
% Census Data	13·4	1·3	5·9	0·2	2·8	18·5	76·3		49·2	50·8
% of NIH-funded TBI trials not reporting demographics	64·4	84·4	84·4	88·9	84·4	80·0	42·2	62·2	17·8	17·8

Race, ethnicity, and gender demographics are reported as percentage. STD is the standard deviation. General population and TBI statistics are from the Census or CDC, respectively.

A majority of the TBI studies did not include racial or ethnic demographics. Most studies did report gender and overwhelmingly male participants (68%). 64·4% of NIH-funded TBI clinical trials did not report or recruit any black subjects and this number was even higher for other races and the Hispanic ethnicity. We analyzed demographic statistics from all reporting TBI clinical trials, determined the number of participants by race, ethnicity, and gender, and calculated the percent from the total number of participants. Our analysis shows that 9.9% of enrolled TBI clinical trial subjects were black people but 19% of mortalities occur among that racial group (14). White people represented 65·3% of study participants but only 52.0% of mortalities.

### Factors potentially impacting study enrollment

Our analysis found that participants of TBI clinical trials require on average 12 days of commitment. Many TBI clinical trials also required phone calls for follow up visits and specifically excluded non-English speakers. Clinical trials that require significant time (12 days) and numerous follow up sessions may exacerbate systemic inequities (transportation issues, pregnancy, childcare, access to healthcare) found by women and people of color.

## Discussion

### Inequities in clinical trial participants and surgical workforce diversities contribute to two-fold increase in the death rate of minorities

Despite advances by women and people of color in the medical field, and the increasing attention to how these factors impact the quality of healthcare, significant disparities persist with regards to brain injury. The consequences of these inequities are grave. The status quo is that people of color are approximately twice as likely as white people to die from brain injury. Our analysis suggests that current hierarchies for funding and conducting research in brain injury are not mitigating the problem. They reveal the stark need to rethink many aspects of how we conduct research and how this translates into the everyday care we provide for our patients.

While the bias toward white males recruited and enrolled in TBI clinical trials may be partially accounted for by subjects' willingness to engage with the healthcare system and attend follow-up appointments, other factors must also be addressed.

Many of the problems contributing to inequity for brain injury are common to other conditions in healthcare but are exacerbated by a field that has uniquely fewer objective measures, greater opportunity for financial inequity to impact outcome, and far less diversity.

### Healthcare inequities are caused by lack of objective measures despite numerous on-going clinical trials

Our review of all clinical trials in traumatic brain injury revealed inclusion of patients in clinical trials is often predicated on classification of injury severity with the Glasgow Coma (GCS) Scale score. A recent NASEM report focused on the GCS as an important classifier for brain injury (25). One potential point at which bias in the quality of care is introduced is pre- hospital and at the level of triage, which is prior to being seen by neurosurgery. Minority individuals presenting with impaired mental status or documented decreased GCS might more often have their altered examination falsely attributed to intoxication or cultural differences rather than brain injury, leading to delays in recognition of brain injury. **To mitigate this inequity, one might contemplate incorporation of a triage system that incorporates more objective measures, rather than GCS.** Patients that are not awake might undergo pupillometry, which is currently under investigation as a triage tool, and an immediate serum marker analysis as numerous studies now demonstrate this to be an accurate means of identifying vascular injury in the brain though it has not been validated for triage (20–22). Patients who are awake could undergo a rapid automated digital neurological examination (26–29). Patients who are awake could undergo a rapid automated digital neurological examination including assessment of the cranial nerves, such as afforded by an eye tracking system that assesses cranial nerves 2 through 7, their nuclei and inputs *via* pupillometry, ocular motility and blink (30–33). Regardless of the mental status of the patient, standard criteria for obtaining a CT scan should be reassessed to ensure lack of bias (34, 35). Clinical trials to ensure the efficacy and objectivity of objective screening measures in a diverse population are warranted prior to wide utilization as it is critical to avoid introduction of objective measures that still perpetuate systemic inequalities. We do not yet know if serum markers, pupillometry or eye tracking are as accurate in people of color as they are in white people.

Incorporation of objective measures for brain injury has the potential to alter the status quo. Imagine the hypothetical scenario of a patient arriving in an emergency department with brain trauma. The perfect scenario is clairvoyant prognostication: a surgeon is able to predict who will have a wretched prognosis regardless of intervention and therefore they do not operate on those destined to be futile. The surgeon will also be able to predict who will benefit from an operation and thus does not risk morbidity/mortality by doing an operation that is theoretically unnecessary. In reality, prognostication can be difficult. A surgeon may overestimate the likelihood of inevitable death, and in predicting a poor outcome ultimately causes it to become inevitable by failing to intervene. Conversely, a surgeon may fail to predict a poor outcome and offer a surgery that ultimately results in survival with a persistent disabling deficit. Another undesirable scenario is that a surgeon may subject a patient who ultimately might have survived without surgery to an unnecessary procedure to avoid missing any of the patients who would benefit from surgery, risking unnecessary morbidity or mortality in that patient. Imposition of physician biases and expectations about patient outcomes on this rubric exacerbates inequity.

Uncomfortable as it may be to acknowledge, the “aggressiveness” of some healthcare personnel in evaluating these patients might be a function of how they perceive that patient will do - their expected mortality, their quality of life, their likelihood of being a burden on society, and their capacity to contribute if disabled by a potentially highly morbid brain injury. It has been established that people with lower education level/socioeconomic status and non-white race are more likely to have poor outcomes after brain injury. **Thus, implicit bias and “ableism” may render surgeons less likely to operate on people who are perceived to be less educated, disabled, poor, or minorities, as they will be more likely to have a poor outcome.** Such factors may be difficult to assess in a trauma situation, and sadly, poor prognostication of brain injury becomes a self-fulfilling prophecy. These implicit biases may be more likely to be perpetuated by a workforce that is culturally dissonant with the patients they are treating. The subconsciously racist, sexist, ableist and classist surgeon may be particularly susceptible to bias.

### Can artificial intelligence or advanced automation correct these inequities?

How can this inequity be corrected? We would argue that a necessary first step to correct the problem is by building accurate prognostication algorithms that are objective and agnostic to language, race, wealth, disability, or education. Such algorithms will likely require a combination of physiologic, molecular, and radiographic measures. Further development of these algorithms could potentially reduce implicit biases in the management of brain injury and improve outcomes for all patients, although great care must be taken to make sure that the algorithms are themselves not biased. Examples of assessors to include in these prognosticating algorithms include measures of brainstem function such as pupillometry, eye tracking or other quantitative cranial nerve function (30–33), serum markers (26), and image analysis (36–39). These are measures that should potentially be able to be confirmed as “colorblind.” Volitional assessments that rely on physician bias, level of patient education, cooperation, absence of cultural dissonance and language skills will likely contribute to inequity.

The utilization of objective measures with machine learning (artificial intelligence) has the potential to reduce inequities in the neurosurgical field through automation, improved accuracy, speed, accessibility, and reduced costs (34–36). A major caveat is that we need to ensure that data elements incorporated into future algorithms do not perpetuate inequity (40–42). Yet, we find the implicit bias currently found in healthcare is further propagated by machine learning due to systemic inequities. An example is that current pulse-oximeters are less accurate in people with dark skin and regulation does not exist to ensure equitable manufacturing of medical devices (43). Gender inequalities in TBI research are multiple such as a standard exclusion factor of pregnancy, nearly all studies focused on males due to increase frequency of head injuries, and overall lack of female-focused therapies. The use of machine learning should eliminate bias and standardize research outcomes (44–46), however, the aforementioned inequities that exist in medical technological and the overwhelming gender and racial bias that currently exists in TBI clinical research datasets (47, 48), produces a perpetual cycle of healthcare improving outcomes for white men but not necessarily for women and people of color.

The National Institutes of Health and other funding agencies with a vested interest in more equitable care should make the funding of research investigating unbiased objective measures for triage and prognostication algorithms a priority to promote equitable outcomes in brain injury.

### Insurance reimbursements for brain injuries causes surgeon burnout and bias toward white males’ patient recruitment

The structure of the American healthcare system is such that insurance companies, Medicare and Medicaid currently reimburse at higher levels for human pathologies that can be objectively measured and are treated with surgery or technologies that rely on device or pharmaceutical intervention. Because brain injury may not always be apparent on conventional imaging, and the lack of objective measures makes it difficult to evaluate and validate therapeutics, financial reimbursement is poor. The ramifications of poor reimbursement include de-prioritization by clinical healthcare systems and increased out-of-pocket costs that ultimately lead to better outcomes in people who can afford to pay for care beyond what insurance will provide. Increased validation of objective measures for injury begins with reimbursement, and eventually will result in effective therapeutics.

Despite the extraordinarily high volume and cost to society of morbidity and mortality from TBI in the U.S., neurotrauma as a specialty is underserved and often not considered as desirable as many of the other neurosurgical specialties. Some of this relates to compensation and some relates to the emergency nature of the work that can impact career satisfaction and burnout. These are challenging economic issues that will likely require legislative intervention to solve.

### Correcting the lack of diversity in the neurosurgical workforce requires systemic change

Women and minorities are under-represented in medicine at progressively disproportionate levels, while white men from wealthy backgrounds are most likely to matriculate into medical school (49). Efforts must be made to correct this inequity. Linkage of national neurosurgical program ranking (48), residency accreditation or ACGME (American College of Graduate Medical Education) (49) funding to hiring, retention and promotion of female and minority residents and faculty might improve these percentages. In addition, since a majority of women and minorities who leave academia likely do so without addressing the problems that drove them out due to fear of retaliation or other adverse consequences (50), organized neurosurgery might consider developing a confidential and anonymous “exit interview” mechanism to identify problems that might be corrected in the future. Finally, the National Institutes of Health should execute its proposed strategy (51) for improving minority participation in research, ensuring that projects proposed by minorities are mentored into funding, and that women and people of color are studied at ratios representative of their likelihood of injury.

Reduction of clinical trial burden (time, number of visits) might make participation for minorities more viable. At a minimum, all NIH funded studies should be disclosing the racial and gender distribution of their research subjects. In addition, the NIH should alter their methods for classifying race and gender as many people have mixed race or binary gender and may be unsure which box to check. Racial and gender inequities in healthcare need to be scrutinized and studied, yet limited research exists on the connections between health insurance reimbursement, socio-economic status and patient outcomes after injury. Research funding must be made available to understand the current state of health care inequities and overcome the bias caused by unfair health insurance practices.

The exodus of women and minorities from healthcare has been described as burnout (52), as moral injury (53), and as death by 1,000 papercuts (54). We would argue that the reason some women or minorities might leave is that they see the status quo, they try to change it and develop the sensation of screaming into a void as the obstacles they encounter are rooted in hierarchical structures and financial hurdles that are insurmountable. Women and minorities in healthcare may work harder and engage in status leveling (55) but are paid less than white men for the same work (56), are harassed more (50), and experience entitling (57), and role incredulity (58). The combination of these injustices along with being asked to be complicit in a system that gaslights and does not provide the same standard of care for all members of society (59) may be morally unconscionable to some women and minorities and potentially impact their decision to leave healthcare.

## Conclusions

The neurosurgical workforce is overwhelmingly white and male. The consequences of this workforce inequity is felt most strongly in a field such as brain injury, which lacks objective measures and classification schemes. Lack of diversity in clinical research teams from leadership to medical students continually perpetuates the inequities engrained into healthcare. It is much harder for minorities to be promoted or receive recognition due to this. Further, the lack of diversity creates implicit bias in clinical research because the demographics of patients recruited into a clinical trial do not represent the real world. As out analysis shows, this results in unfavorable healthcare for minorities. Outcomes after brain injury are worse for minority and female populations due to systemic inequities in healthcare leadership, research participation and every aspect of patient care from triage to rehabilitation. Multiple strategies are needed to correct these inequities including validation of objective measures for the triage and prognostication of brain injured patients. Development of machine learning and artificial intelligence algorithms may reduce inequity if precautions are taken against the incorporation of measures influenced by race, gender or other factors creating bias. Aspects of the inequities associated with brain injury are common to most of the healthcare system and require fundamental shifts in how healthcare is conducted.

## References

[1] Renfrow JJ Rodriguez A Wilson TA Germano IM Abosch A Wolfe SQ. Tracking career paths of women in neurosurgery. Neurosurgery. (2018) 82(4):576–82. 10.1093/neuros/nyx251 28521026

[2] Detchou DK Onyewuenyi A Reddy V Boyke A Mbabuike N Ashley WW Letter: a call to action: increasing black representation in neurological surgery. Neurosurgery. (2021) 88(5):E469–73. 10.1093/neuros/nyab057 33611592 PMC8046586

[3] AAMC. Diversity in Medicine: Facts and Figures 2019. (2019).

[4] Lynch G Nieto K Puthenveettil S Reyes M Jureller M Huang JH. Attrition rates in neurosurgery residency: analysis of 1361 consecutive residents matched from 1990 to 1999. J Neurosurg. (2015) 122(2):240–9. 10.3171/2014.10.JNS132436 25415065

[5] Kim EE Klein AL Lartigue JW Hervey-Jumper SL Rosseau G. Diversity in neurosurgery. World Neurosurg. (2021) 145:197–204. 10.1016/j.wneu.2020.08.219 32891852 PMC7470761

[6] Linton KF Kim BJ. Traumatic brain injury as a result of violence in Native American and black communities spanning from childhood to older adulthood. Brain Inj. (2014) 28(8):1076–81. 10.3109/02699052.2014.901558 24702677

[7] Oh SS Galanter J Thakur N Pino-Yanes M Barcelo NE White MJ. Diversity in clinical and biomedical research: a promise yet to be fulfilled. PLoS Med. (2015) 12(12):e1001918. 10.1371/journal.pmed.1001918 26671224 PMC4679830

[8] Betz ME Li G. Epidemiologic patterns of injuries treated in ambulatory care settings. Ann Emerg Med. (2005) 46(6):544–51. 10.1016/j.annemergmed.2005.07.009 16308072

[9] Peterson AB Sarmiento K Xu L Haileyesus T. Traumatic brain injury-related hospitalizations and deaths among American Indians and Alaska natives - United States, 2008–2014. J Safety Res. (2019) 71:315–8. 10.1016/j.jsr.2019.09.017 31862042

[10] Bazarian JJ Pope C McClung J Cheng YT Flesher W. Ethnic and racial disparities in emergency department care for mild traumatic brain injury. Acad Emerg Med. (2003) 10(11):1209–17. 10.1197/S1069-6563(03)00491-3 14597497

[11] Fuentes MM Jimenez N Apkon SD Rivara FP. Functional outcomes during inpatient rehabilitation for American Indian and Alaska Native children with traumatic brain injury. J Pediatr Rehabil Med. (2016) 9(2):133–41. 10.3233/PRM-160376 27285806 PMC5099074

[12] Corrigan JD Harrison-Felix C Bogner J Dijkers M Terrill MS Whiteneck G. Systematic bias in traumatic brain injury outcome studies because of loss to follow-up. Arch Phys Med Rehabil. (2003) 84(2):153–60. 10.1053/apmr.2003.50093 12601644

[13] Krellman JW Kolakowsky-Hayner SA Spielman L Dijkers M Hammond FM Bogner J. Predictors of follow-up completeness in longitudinal research on traumatic brain injury: findings from the National Institute on Disability and Rehabilitation Research traumatic brain injury model systems program. Arch Phys Med Rehabil. (2014) 95(4):633–41. 10.1016/j.apmr.2013.10.016 24211496

[14] Miller GF Daugherty J Waltzman D Sarmiento K. Predictors of traumatic brain injury morbidity and mortality: examination of data from the national trauma data bank: predictors of TBI morbidity & mortality. Injury. (2021) 52(5):1138–44. 10.1016/j.injury.2021.01.042 33551263 PMC8107124

[15] Whaley CM Koo T Arora VM Ganguli I Gross N Jena AB. Female physicians earn an estimated $2 million less than male physicians over A simulated 40-year career. Health Aff. (2021) 40(12):1856–64. 10.1377/hlthaff.2021.00461 PMC9910787 34871074

[16] Abosch A Rutka JT. Women in neurosurgery: inequality redux. J Neurosurg. (2018) 129(2):277–81. 10.3171/2018.4.JNS172878 29999441

[17] Shin AY. The color of surgery. Tech Hand Up Extrem Surg. (2020) 24(3):107. 10.1097/BTH.0000000000000309 32701772

[18] Abelson JS Wong NZ Symer M Eckenrode G Watkins A Yeo HL. Racial and ethnic disparities in promotion and retention of academic surgeons. Am J Surg. (2018) 216(4):678–82. 10.1016/j.amjsurg.2018.07.020 30086831

[19] Dawes DE Dunlap NJ Johnson SM. The Surgeon's role in addressing racism and achieving health equity. Am Surg. (2021) 87(11):1704–12. 10.1177/00031348211038562 34412516

[20] Coronado VG Xu L Basavaraju SV McGuire LC Wald MM Faul MD Surveillance for traumatic brain injury-related deaths– United States, 1997–2007. MMWR Surveill Summ. (2011) 60(5):1–32. 21544045

[21] Daugherty J Waltzman D Sarmiento K Xu L. Traumatic brain injury-related deaths by race/ethnicity, sex, intent, and mechanism of injury - United States, 2000–2017. MMWR Morb Mortal Wkly Rep. (2019) 68(46):1050–6. 10.15585/mmwr.mm6846a2 31751321 PMC6871899

[22] Brenner EK Grossner EC Johnson BN Bernier RA Soto J Hillary FG. Race and ethnicity considerations in traumatic brain injury research: incidence, reporting, and outcome. Brain Inj. (2020) 34(6):799–808. 10.1080/02699052.2020.1741033 32228303

[23] Bowman SM Martin DP Sharar SR Zimmerman FJ. Racial disparities in outcomes of persons with moderate to severe traumatic brain injury. Med Care. (2007) 45(7):686–90. 10.1097/MLR.0b013e31803dcdf3 17571018

[24] Iskander JK Crosby AE. Implementing the national suicide prevention strategy: time for action to flatten the curve. Prev Med. (2021) 152(Pt 1):106734. 10.1016/j.ypmed.2021.106734 34344523 PMC8443844

[25] National Academies of Sciences E, Medicine. Traumatic brain injury: A roadmap for accelerating progress. Washington, DC: The National Academies Press (2022). 35533242

[26] Mahan MY Thorpe M Ahmadi A Abdallah T Casey H Sturtevant D Glial fibrillary acidic protein (GFAP) outperforms S100 calcium-binding protein B (S100B) and ubiquitin C-terminal hydrolase L1 (UCH-L1) as predictor for positive computed tomography of the head in trauma subjects. World Neurosurg. (2019) 128:e434–44. 10.1016/j.wneu.2019.04.170 31051301

[27] Castaño-Leon AM Sánchez Carabias C Hilario A Ramos A Navarro-Main B Paredes I Serum GFAP and UCH-L1 for prediction of absence of intracranial injuries on head CT (ALERT-TBI): a multicentre observational study. Lancet Neurol. (2018) 17(9):782–9. 10.1016/S1474-4422(18)30231-X 30054151

[28] Welch RD Ayaz SI Lewis LM Unden J Chen JY Mika VH. Ability of serum glial fibrillary acidic protein, ubiquitin C- terminal hydrolase-L1, and S100B to differentiate normal and abnormal head computed tomography findings in patients with suspected mild or moderate traumatic brain injury. J Neurotrauma. (2016) 33(2):203–14. 10.1089/neu.2015.4149 26467555 PMC4722555

[29] Cohen AB Nahed BV. The digital neurologic examination. Digit Biomark. (2021) 5(1):114–26. 10.1159/000515577 34056521 PMC8138139

[30] Bin Zahid A Hubbard ME Lockyer J Podolak O Dammavalam VM Grady M Eye tracking as a biomarker for concussion in children. Clin J Sport Med. (2020) 30(5):433–43. 30095503 10.1097/JSM.0000000000000639

[31] Samadani U Farooq S Ritlop R Laska E Ritlop R Kolecki R Detection of third and sixth cranial nerve palsies with a novel method for eye tracking while watching a short film clip. J Neurosurg. (2015) 122(3):707–20. 10.3171/2014.10.JNS14762 25495739 PMC4547625

[32] Samadani U Li M Qian M Sensitivity and specificity of an eye movement tracking-based biomarker for concussion. Concussion. (2016) 1(1):CNC3. 10.2217/cnc.15.2 30202548 PMC6114025

[33] Samadani U Ritlop R Reyes M Eye tracking detects disconjugate eye movements associated with structural traumatic brain injury and concussion. J Neurotrauma. (2015) 32(8):548–56. 10.1089/neu.2014.3687 25582436 PMC4394159

[34] Gerber N Sookraj K Munnangi S Angus LDG Lamba V Kumar K Impact of the Pediatric Emergency Care Applied Research Network (PECARN) guidelines on emergency department use of head computed tomography at a level I safety-net trauma center. Emerg Radiol. (2019) 26(1):45–52. 10.1007/s10140-018-1645-4 30259227

[35] Kwon BS Song HJ Lee JH. External validation and comparison of the Pediatric Emergency Care Applied Research Network and Canadian Assessment of Tomography for Childhood Head Injury 2 clinical decision rules in children with minor blunt head trauma. Clin Exp Emerg Med. (2021) 8(3):182–91. 10.15441/ceem.20.123 34649406 PMC8517464

[36] Mahan MY Rafter DJ Truwit CL Oswood M Samadani U. Evaluation of diffusion measurements reveals radial diffusivity indicative of microstructural damage following acute, mild traumatic brain injury. Magn Reson Imaging. (2021) 77:137–47. 10.1016/j.mri.2020.12.012 33359428

[37] Bin Zahid A Balser D Thomas R Mahan MY Hubbard ME Samadani U. Increase in brain atrophy after subdural hematoma to rates greater than associated with dementia. J Neurosurg. (2018) 129(6):1579–87. 10.3171/2017.8.JNS17477 29498578

[38] Mahan M Rafter D Casey H Engelking M Abdallah T Truwit C Tbiextractor: a framework for extracting traumatic brain injury common data elements from radiology reports. PLoS One. (2020) 15(7):e0214775. 10.1371/journal.pone.0214775 32609723 PMC7329124

[39] Bin Zahid A Mikheev A Srivatsa N Babb J Samadani U Rusinek H. Accelerated brain atrophy on serial computed tomography: potential marker of the progression of Alzheimer disease. J Comput Assist Tomogr. (2016) 40(5):827–32. 10.1097/RCT.0000000000000435 27224227 PMC5025331

[40] Zhao C Huang WJ Feng F Zhou B Yao HX Guo YE Abnormal characterization of dynamic functional connectivity in Alzheimer's Disease. Neural Regen Res. (2022) 17(9):2014–21. 10.4103/1673-5374.332161 35142691 PMC8848607

[41] Collins GS Dhiman P Andaur Navarro CL Ma J Hooft L Reitsma JB Protocol for development of a reporting guideline (TRIPOD-AI) and risk of bias tool (PROBAST-AI) for diagnostic and prognostic prediction model studies based on artificial intelligence. BMJ Open. (2021) 11(7):e048008. 10.1136/bmjopen-2020-048008 34244270 PMC8273461

[42] Chrusciel J Girardon F Roquette L Laplanche D Duclos A Sanchez S. The prediction of hospital length of stay using unstructured data. BMC Med Inform Decis Mak. (2021) 21(1):351. 10.1186/s12911-021-01722-4 34922532 PMC8684269

[43] Okunlola OE Lipnick MS Batchelder PB Bernstein M Feiner JR Bickler PE. Pulse oximeter performance, racial inequity, and the work ahead. Respir Care. (2022) 67(2):252–7. 10.4187/respcare.09795 34772785

[44] Tariciotti L Palmisciano P Giordano M Remoli G Lacorte E Bertani G Artificial intelligence-enhanced intraoperative neurosurgical workflow: current knowledge and future perspectives. J Neurosurg Sci. (2022) 66(2):139–50. 10.23736/S0390-5616.21.05483-7 34545735

[45] Lee MS Guo LN Nambudiri VE. Towards gender equity in artificial intelligence and machine learning applications in dermatology. J Am Med Inform Assoc. (2022) 29(2):400–3. 10.1093/jamia/ocab113 34151976 PMC8757299

[46] Lupei MI Li D Ingraham NE Baum KD Benson B Puskarich M A 12-hospital prospective evaluation of a clinical decision support prognostic algorithm based on logistic regression as a form of machine learning to facilitate decision making for patients with suspected COVID-19. PLoS One. (2022) 17(1):e0262193. 10.1371/journal.pone.0262193 34986168 PMC8730444

[47] Suri JS Bhagawati M Paul S Protogeron A Sfikakis PP Kitas GD Understanding the bias in machine learning systems for cardiovascular disease risk assessment: the first of its kind review. Comput Biol Med. (2022) 142:105204. 10.1016/j.compbiomed.2021.105204 35033879

[48] Monlezun DJ Samura AT Patel RS Thannoun TE Balan P. Racial and socioeconomic disparities in out-of-hospital cardiac arrest outcomes: artificial intelligence-augmented propensity score and geospatial cohort analysis of 3,952 patients. Cardiol Res Pract. (2021) 2021:3180987. 10.1155/2021/3180987 34868674 PMC8635948

[49] Shahriar AA Puram VV Miller JM Sagi V Castañón-Gonzalez LA Prasad S Socioeconomic diversity of the matriculating US medical student body by race, ethnicity, and sex, 2017–2019. JAMA Network Open. (2022) 5(3):e222621. 10.1001/jamanetworkopen.2022.2621 35289863 PMC8924719

[50] Benzil DL Muraszko KM Soni P Air EL Orrico KO Rutka JT. Toward an understanding of sexual harassment in neurosurgery. J Neurosurg. (2020):1–10. 10.3171/2020.6.JNS201649 33171438

[51] Armstrong K Ritchie C. Research participation in marginalized communities - overcoming barriers. N Engl J Med. (2022) 386(3):203–5. 10.1056/NEJMp2115621 35029846

[52] Jackson TN Pearcy CP Khorgami Z Agrawal V Taubman KE Truitt MS. The physician attrition crisis: a cross-sectional survey of the risk factors for reduced job satisfaction among US surgeons. World J Surg. (2018) 42(5):1285–92. 10.1007/s00268-017-4286-y 29067517

[53] PTSD PA-CfpmhatCcoeo. Racial inequities and moral distress: a supplement to moral stress amongst healthcare workers during COVID-19. The Moral Injury Guide (2021).

[54] Goldberg E. For Doctors of Color, Microaggressions Are All Too Familar. New York Times. (2020).

[55] Cardador MT Hill PL Salles A. Unpacking the Status-leveling burden for women in male-dominated occupations. Adm Sci Q. (2021) 67(1):237–84.

[56] Rimmer A O'Dowd A. Women doctors paid less than men even after part time working is accounted for. Br Med J. (2020) 371:m4904. 10.1136/bmj.m4904 33334729

[57] Diehl AaDL. We need to stop “untitling” and “uncredentialing” professional women. New York, NY: Fast Company (2021).

[58] Diehl AaDL. When people assume you’re not in charge because you’re a woman. Boston, MA: Harvard Business Review (2021).

[59] Fraser S. The toxic power dynamics of gaslighting in medicine. Can Fam Physician. (2021) 67(5):367–8. 10.46747/cfp.6705367 33980633 PMC8115954

